# Clinical Efficacy and Potential Mechanisms of Acupoint Stimulation Combined With Chemotherapy in Combating Cancer: A Review and Prospects

**DOI:** 10.3389/fonc.2022.864046

**Published:** 2022-04-25

**Authors:** Shanshan Li, Suhong Zhao, Yi Guo, Yuanzhen Yang, Jin Huang, Jiaqi Wang, Shanshan Lu, Bin Wang, Chao Chai, Zhifang Xu, Yenlie Chin

**Affiliations:** ^1^ Research Center of Experimental Acupuncture Science, Tianjin University of Traditional Chinese Medicine, Tianjin, China; ^2^ School of Traditional Chinese Medicine, Tianjin University of Traditional Chinese Medicine, Tianjin, China; ^3^ National Clinical Research Center for Chinese Medicine Acupuncture and Moxibustion, Tianjin, China; ^4^ Tianjin Medical University Cancer Institute and Hospital, National Clinical Research Center for Cancer, Key Laboratory of Cancer Prevention and Therapy, Tianjin’s Clinical Research Center for Cancer, Tianjin, China; ^5^ Department of Radiology, Tianjin Institute of Imaging Medicine, Tianjin First Central Hospital, School of Medicine, Nankai University, Tianjin, China; ^6^ School of Acupuncture and Moxibustion and Tuina, Tianjin University of Traditional Chinese Medicine, Tianjin, China

**Keywords:** acupoint stimulation, chemotherapy, cancer, neuroimmune, acupuncture

## Abstract

Although chemotherapy is the first-line treatment strategy for a variety of tumors, its side effects have limited its efficacy. This review summarizes the progress on the use of acupoint stimulation to combat chemotherapy-associated side effects, including chemotherapy-induced peripheral neuropathy (CIPN), cognitive impairment (CICI), and gastrointestinal toxicity (GI), as well as myelosuppression and immunosuppression. It was found that acupoint stimulation attenuated CIPN and GI by modulating the 5-hydroxytryptamine system in dorsal root ganglia, the dorsal horn of the spinal cord, and the duodenum by reducing oxidative stress and neuroinflammation. Acupoint stimulation also alleviated GI by activating vagal activity in the nucleus tractus solitarius and promoting the secretion of gastrointestinal neuropeptide hormones. Acupoint stimulation restored both bone marrow hematopoiesis and immune function to combat cancer. In addition, the combination of acupoint stimulation and chemotherapy could inhibit tumor growth by promoting tumor cell apoptosis and the enrichment of chemotherapeutic agents in tumor tissue and by modulating the tumor immune microenvironment and normalizing the vasculature. Multiple evidence also indicates that neuroimmune regulation may be involved in the effects of acupoint stimulation. In conclusion, the evidence suggests that acupoint stimulation can alleviate the side effects of chemotherapy and can also assist chemotherapeutic agents in inhibiting tumor growth, which expands the clinical application of acupoint stimulation in cancer treatment. However, more high-quality clinical studies are needed to confirm the clinical value of acupoint stimulation.

## Introduction

Cancer, as a major public health problem worldwide, is one of the principal causes of death in both developed and developing countries. Many countries face huge challenges in managing the increasing burden of cancer (1). Chemotherapy has been demonstrated to be effective against a variety of cancers including breast cancer, non-small cell lung cancer (NSCLC), colon cancer, and others (2–4). It is also used as a first-line treatment strategy for many cancer patients with inoperable disease, advanced metastasis, and poor immunotherapy results. Although chemotherapeutic agents can inhibit cancer cell proliferation and kill cancer cells, they also attack benign/normal cells (5), leading to a wide range of side effects and reducing the long-term efficacy of the treatment (6). Chemotherapeutic agents may damage many systems in the body, including the nervous system [chemotherapy-induced peripheral neuropathy (CIPN); chemotherapy-induced cognitive impairment (CICI)], digestive system [chemotherapy-induced nausea and vomiting (CINV); chemotherapy-induced anorexia (CIA)], and the hematopoietic and immune systems (myelosuppression, anemia, and immune deficiency). Although some side effects can be alleviated to a certain extent after pharmacological intervention, many cannot. This limits the anticancer efficacy of chemotherapy. For instance, antidepressants, anticonvulsants such as gabapentin, and narcotic and non-narcotic analgesics are currently used to treat neurotoxicity in cancer patients; although only duloxetine is currently recommended for CIPN pain, it cannot relieve paresthesia. Additionally, these medications may cause side effects such as fatigue, insomnia, or nausea (7). Thus, the prevention and mitigation of various side effects of cancer chemotherapy are key to improving the clinical efficacy of cancer chemotherapy.

Acupuncture, as a common acupoint stimulation method, has been approved for use in 183 countries and states. The World Health Organization has recommended 107 indications for acupuncture, including various disorders of the nervous system (8), musculoskeletal system (9), respiratory system (10), and digestive system (11). Besides, various acupoint stimulation modalities such as electroacupuncture (EA), transcutaneous electrical acupoint stimulation (TEAS), bee venom acupuncture therapy (BVA), and laser acupuncture (LA) have been used for the management of various chemotherapy side effects. For instance, a retrospective cohort study showed that acupoint stimulation improved the symptoms of chemotherapy-induced myelosuppression, including low white blood cell counts (12). Similarly, another meta-analysis that included 12 randomized controlled trials (RCT) also reported that acupressure protected against CINV (13). More recently, it was found that patients with stage IIb–IIIb cervical squamous cell carcinoma, who had received four cycles of cisplatin combined with EA treatment, had both increased numbers of natural killer (NK) cells in their peripheral blood and reduced tumor volumes (14). Thus, acupoint stimulation can mitigate the side effects and potentiate the efficacy of chemotherapy.

Here, we have reviewed 33 studies published in the past 10 years on the effects and mechanisms of acupoint stimulation on reducing the toxicity and enhancing the antitumor efficacy of chemotherapy in cancer patients and animal models. Articles were identified in the PubMed database. We also discuss the potential mechanisms that are likely to be involved in the effects of acupoint stimulation. This work may provide a basis for future prospects on the application of acupoint stimulation in the treatment of cancer and chemotherapy side effects.

## Efficacy and Mechanisms of Acupoint Stimulation in the Alleviation of Tumor Chemotherapy-Induced Side Effects

The 26 studies in which acupoint stimulation was found to ameliorate chemotherapy side effects focused on neurotoxicity including CIPN and CICI, gastrointestinal toxicity (GI) including CINV and CIA, and myelosuppression and immunosuppression. A variety of acupoint stimulation methods were used in these studies including acupuncture, TEAS, EA, LA, combined acupuncture and moxibustion, or combined acupuncture and EA. ST36 was commonly used to treat a variety of side effects after tumor chemotherapy such as peripheral nerve injury (15, 16), nausea and vomiting, anorexia (17), and myelosuppression and immunosuppression (18).

According to the theory of acupuncture and moxibustion in traditional Chinese medicine, acupoints on different meridians have different functions, and stimulation on an acupoint located in the vicinity of a lesion is considered to treat the lesion. In clinical applications, *Taichong* (LR3), *Zusanli* (ST36), and *Sanyinjiao* (SP6) were commonly used for treating CIPN and CICI. CIPN is mainly characterized by abnormal sensory function in the limbs, and seven clinical studies showed that the majority of the selected acupoints were located in the upper and lower extremities. These included LR3, ST36, SP6, and *Hegu* (LI4). *Neiguan* (PC6), ST36, and SP6 were usually used for chemotherapy-induced GI, with PC6 being the most frequently used acupoint. ST36, SP6, and LI4 were used for treating myelopoiesis, while ST36 was also used to inhibit tumor growth.

Compared with clinical studies, fewer acupoints were used in basic research. The EA and BVA were used for chemotherapy-induced neurotoxicity. ST36 was the most common acupoint used for CIPN, either alone or in combination with *Kunlun* (BL60) or LI4. In addition, PC6, *Jianshi* (PC5), *Changqiang* (GV1), and *Huantiao* (GB30) were also used for treating CIPN. Of the acupoints selected for chemotherapy-induced GI, *Zhongwan* (CV12) acupoint was the most frequently used, which differs from the acupoint selection in clinical research. In addition, ST36 is more commonly used to mitigate myelosuppression and immunosuppression and to inhibit tumor growth.

### Chemotherapy-Induced Neurotoxicity

Neurotoxicity disorders resulting from cancer-related chemotherapy include CIPN and CICI. CIPN is one of the common and serious side effects of chemotherapy, and it was supposed that it arises from axonopathy caused by axonal damage and dying back in regions involving the dorsal root ganglia (19). The main symptoms include sensory abnormalities, pain, numbness, tingling, and autonomic neuropathy. The appearance of CIPN may lead to reductions in the chemotherapy dose or treatment termination, ultimately affecting overall survival. CICI usually manifests as memory loss, forgetfulness, difficulties in learning, attention, concentration, and coordination of multitasking and organization. Over 75% of cancer patients have been reported to experience acute cognitive symptoms during chemotherapy, with 17%–34% of patients having long-term posttreatment cognitive deficits that can persist for up to 10 years (20). Several cytotoxic drugs, for example, the anti-tubulins (paclitaxel, vincristine), platinum analogs (cisplatin, carboplatin, oxaliplatin), and the cyclophosphamides, can cause both CIPN and CICI (21). In the case of paclitaxel and oxaliplatin, for example, the incidence of peripheral neuropathy was as much as 92% and 96%, respectively (22).

Acupoint stimulation can mitigate peripheral nerve damage and cognitive impairment caused by the use of chemotherapy drugs such as paclitaxel and oxaliplatin. The severity of CIPN symptoms can be classified into five grades from mild to severe, namely, grade 0; mild, grade 1; moderate, grade 2; severe, grade 3; and very severe, grade 4 (23). As shown in **Table 1**, Lu et al. (7) found that compared with conventional care, 8-week acupuncture treatment was more effective in relieving tingling, numbness, and pain in the hands or feet caused by moderate CIPN in breast cancer patients, and the quality of life of patients was significantly improved after acupuncture treatment. This evidence suggests that acupoint stimulation can be effective in relieving moderate CIPN symptoms including pain and numbness induced by paclitaxel. In addition, in gastric cancer patients with moderate or severe CIPN caused by oxaliplatin, LA treatment for 4 weeks effectively improved the symptoms of cold pain and numbness in the upper and lower limbs (24). A randomized controlled pilot study observed significant improvements in the physical and functional domains of the European Organization for the Research and Treatment of Cancer Quality of Life Questionnaire Core Scale after 5 weeks of acupuncture treatment, together with improved results on the National Cancer Institute Common Terminology for Adverse Events Scale and improvements in the neuropathy sensory symptoms of the acupuncture group after treatment, with no adverse events (25). Moreover, it has been shown that acupuncture combined with mecobalamin for 30 days is more effective than mecobalamin alone in reducing pain in patients with moderate or severe CIPN associated with multiple myeloma (26). However, another study found that EA given to patients for 16 weeks at the same time as chemotherapy did not significantly reduce the severity of pain in breast cancer patients treated with paclitaxel (27).

**Table 1:** The actions and mechanisms of acupoint stimulation in chemotherapy-induced neurotoxicity.

Clinical trials													
Ref.	Randomized type of clinical trial	Jadad score /NOS Stars	Clinical condition		Intervention		Comparison		Acupoints		Acupoint stimulation parameters		Outcomes
Lu (7)	Yes, RCT	Jadad, 4	Breast cancer patients		Acupuncture and EA (n = 20)		Usual care (n = 20)		EX-HN3; bilateral: EX-UE9, TE5, SP6, LR3, LI11, SP9, ST36, KI3		Acupuncture: first week; EA: *deqi*, 2–10 Hz, 30 min, 18 times, 2–8 weeks		PNQ sensory symptoms score↓, Fact-Ntx score↓, BPI-SF score↓, quality of life score↑
Ling (24)	No, PCS	NOS, 5	Gastrointestinal cancer patients		LA (n = 17)		Baseline		Bilateral: PC6, PC7, PC8, PC9, LU11, SP6, KI3, BL60, KI1		780 nm, 100 Hz, 0.2 cm^2^, 80 mW, *deqi*, 20 min, 3 times/week, 4 weeks		Quantitative touch-detection threshold↑, cold-triggered pain withdrawal latency↑, PQAS score↓, CINQ score↑, OSNS score↑
D’Alessandro (25)	Yes, RCT	Jadad, 6	CIPN patients		Acupuncture (n = 15)		Rehabilitation (n = 14)		Bilateral: LR3, SP3, KI3, HT7, PC7, LU9, SP9; wrist-ankle technique (areas 1–3)		30 min, 2 times/week, 5 weeks		NCI CTCAE score, EORTC QLQ score, VAS score
Han (26)	Yes, RCT	Jadad, 2	MM patients		Acupuncture (n = 52)		Mecobalamin (n = 52)		GV14, GV12, GV11, GV9; bilateral: LR3, ST43, GB41, SP6, ST36, SP10, ST25, BL13, BL17, BL58		7–25 mm, *deqi*, 30 min, 3 days, 10 days/session, 3 sessions		VAS score↓, Fact/GOG-Ntx score↓
Greenlee (27)	Yes, RCT	Jadad, 5	Breast cancer patients		EA (n = 31)		Sham EA (n = 32)		EX-B2, EX-LE10; bilateral: GB34, ST36, LI4, LI10		3–4 mm, *deqi*, 2 Hz, 30 min/week, 12 weeks		BPI-SF score↑
Tong (28)	Yes, RCT	Jadad, 3	Breast cancer patients		Acupuncture (n = 39)		Control (n = 36)		GV21, EX-HN1; bilateral: KI3		10–15 mm, *deqi*, 30 min, 5 days, 8 weeks		Fact-COG (PCI, QOL, OTH, PCA) ↑, AVLT↑, CDT↑, serum BDNF↑
Zhang (20)	Yes, RCT	Jadad, 5	Breast cancer patients		Acupuncture and EA (n = 46)		MAS (n = 47)		GV26, CV12, CV4, GV20, EX-HN3; bilateral: HT7, LI4, TE5, SP6, ST8, GB8, ST36, ST40, GB15, EX-HN5, EX-HN1		10–30 mm, 6 V, 48 mA, 2 Hz, 100 μs, 30 min, 2 times/week, 8 weeks		MoCA↑, incidences associated with breast cancer and chemotherapy (diarrhea, tenseness, worrisome, irritation, headache, tinnitus) ↓
Basic researches													
Ref.	Model	Chemotherapeutic agents		Intervention		Acupoints		Acupoint stimulation parameters		Efficacy indicators		Mechanism indicators	
Lee (29)	CIPN rats	Oxaliplatin, 6 mg/kg, i.p.		BVA		GV3		BV: 0.25 mg/kg		—		Spinal cord: 5-HT3↑	
Li (30)	CIPN rats	Oxaliplatin, 6 mg/kg, i.p.		BVA		ST36		BV: 1 mg/kg		Cold and mechanical allodynia↓		Action potential and current threshold in acutely dissociated DRG neurons↑	
Zhang (31)	CIPN rats	Paclitaxel, 2 mg/kg, 4 alternate days, i.p.		EA		GB30		2 mA, 10 Hz, 0.4 ms, pulse EA for 30 min, 7 alternate days		Mechanical allodynia↓, hyperalgesia↓, mechanical responses to stimulation		Spinal cord: p-CaMKII↓	
Li (32)	CIPN rats	Paclitaxel, 24 mg/kg, 2 days apart, i.p.		EA		ST36, BL60		2 Hz, square wave current output (pulse width: 0.2 ms); Intensities ranging from 0.5 to 1.5 mA (increased by 0.5 mA every 10 min, for a total of 30 min) were delivered for a period of 30 min		Thermal↓, mechanical hypersensitivities↓		DRGs (TLR4↓, MyD88↓, TRPV1↓), peak amplitude of the Ca2+ traces, AUC of the Ca2+ traces↓, astrocyte and microglia activation in SCDH↓	
Zhao (33)	CIPN rats	Paclitaxel 1 mg/kg/one injection in 0.5 ml of volume, every 2 days (four injections), i.p.		EA		PC6, PC5		2 mA, 2 Hz, 0.5 ms/pulses, 20 min/day for 5–7 days before each of the experiments		Mechanical and thermal sensitivity↓		DRG: Nrf2↓, NQO1↓, SOD↓, NOX4↓, IL-1β↓, IL-6↓, TNF-α↓	
Choi (34)	CIPN rats	Paclitaxel,2 mg/ml,4 alternate days, i.p.		BVA		LI11, ST36		BV: 1 mg/kg		Mechanical and thermal sensitivity↓, hyperexcitation in WDR neurons↓		—	

↑, upregulated by intervention; ↓, downregulated by intervention.

RCT, randomized controlled trial; EX-HN3, Yintang; EX-UE9, Baxie; TE5, Waiguan; SP6, Sanyinjiao; LR3, Taichong; LI11, Quchi; SP9, Yinlingquan; ST36, Zusanli; KI3, Taixi; EA, electroacupuncture; PNQ, patient neurotoxicity questionnaire; Fact-Ntx, functional assessment of cancer therapy-taxane neurotoxicity subscale; BPI-SF, brief pain inventory-short form; PCS, pilot prospective cohort study; LA, laser acupuncture; PC6, Neiguan; PC7, Daling; PC8, Laogong; PC9, Zhongchong; LU11, Shaoshang; BL60, Kunlun; KI1, Yongquan; PQAS, pain quality assessment scale; CINQ, chemotherapy-induced neurotoxicity questionnaire; OSNS, oxaliplatin-specific neurotoxicity scale; CIPN, chemotherapy-induced peripheral neuropathy; SP3, Taibai; HT7, Shenmen; LU9, Taiyuan; NCI CTCAE, National cancer institute common terminology criteria for adverse events; EORTC QLQ, European organisation for the research and treatment of cancer quality of life questionnaire core; VAS, visual analogue scale; MM, multiple myeloma; GV14, Dazhui; GV12, Shenshu; GV11, Shendao; GV9, Zhiyang; ST43, Xiangu; GB41, Zulinqi; SP10, Xuehai; ST25, Tianshu; BL13, Feishu; BL17, Geshu; BL58, Feiyang; EA, electroacupuncture; EX-B2, Huatuojiaji; EX-LE10, Bafeng; GB34, Yanglinquan; LI10, Shousanli; GV21, Qianding; EX-HN1, Sishencong; Fact-COG, functional assessment of cancer treatment cognition test; PCI, perceived cognitive impairments; QOL, impact on quality of life; OTH, comments from others; PCA perceived cognitive abilities; AVLT, auditory verbal learning test; CDT, clock-drawing test; BDNF, brain-derived neurotrophic factor; MAS, minimal acupuncture stimulation; GV26, Shuigou; CV12, Zhongwan; CV4, Guanyuan; GV20, Baihui; ST8, Touwei; GB8, Shuaigu; GB15, Toulinqi; EX-HN5, Taiyang; MoCA, montreal cognitive assessment; BVA, bee venom acupuncture; GV3, Yaoyangguan; 5-HT3, 5-hydroxytryptamine type 3; DRG, dorsal root ganglion; GB30, Huantiao; p-CaMKII, phosphorylated calcium/calmodulin-dependent protein kinase II; TLR4, toll-like receptor 4; MyD88, myeloid differentiation primary response protein 88; TRPV1, transient receptor potential vanilloid 1; AUC, area under the curve; SCDH, spinal cord dorsal horn; PC5, Jianshi; Nrf2, nuclear factor (erythroid-derived 2)-like 2; NQO1, NADPH quinone oxidoreductase-1; SOD, superoxide dismutases; NOX4, NADPH oxidase 4; IL-1β, interleukin-1β; IL-6, interleukin-6; TNF-α, tumor necrosis factor-alpha; WDR, wide dynamic range; NOS, Newcastle–Ottawa Scale.

In conclusion, most evidence suggests that interventions with multiple acupoint stimulation can be effective in preventing and treating moderate or severe CIPN symptoms (such as neuropathic pain and numbness) induced by paclitaxel or oxaliplatin and that acupoint stimulation is effective as adjuvant therapy for pain reduction. Nevertheless, more high-quality evidence is needed to explore the clinical value of acupoint stimulation.

Considering CICI, an RCT including 80 breast cancer patients treated with a paclitaxel-based chemotherapy regimen reported that acupuncture treatment lasting for 8 weeks was effective in improving memory, learning, and executive functioning. It is possible that its therapeutic effects may be associated with the regulation of brain-derived neurotrophic factor (BDNF) (28). Zhang et al. (20) gave EA trigeminal nerve stimulation combined with body acupuncture for 8 weeks to breast cancer patients who had received cyclophosphamide and found that EA combined with body acupuncture could improve reverse digit breadth test (evaluation of attentional function and working memory) scores and decrease the incidence of diarrhea, loss of appetite, headache, and anxiety when compared with patients in the low-frequency EA group. This suggested that acupoint stimulation was not only effective in improving the symptoms of CICI but may also reduce gastrointestinal symptoms, headache, and anxiety.

To date, basic research has focused mainly on how BVA and EA therapy improves CIPN. The study designs mostly used oxaliplatin (29, 30) and paclitaxel (31–34) as interventions in healthy rats. The symptoms of oxaliplatin-induced neuropathy were essentially divided into two types: acute onset of cold hypersensitivity and chronic sensory neuropathy including abnormal tactile pain and numbness. As shown in **Table 1**, Lee et al. (29) created a model of acute cold pain induced by a single intraperitoneal injection of oxaliplatin in healthy rats, followed by subcutaneous injection of BVA into *Yaoyangguan* (GV3), finding that BVA relieved oxaliplatin-induced cold pain in the rats. Elevated levels of serotonin and 5-hydroxytryptamine (5-HT) were also detected in the spinal cords of the BVA-treated rats. It was also found that depletion of serotonin with DL-p-chlorophenylalanine or 5-HT receptor antagonists eliminated BVA alleviation of oxaliplatin-induced cold allodynia, confirming that BVA treatment attenuated 5-HT type-3 receptor-mediated oxaliplatin-induced cold pain by activating the serotonergic system. Furthermore, BVA at another acupoint ST36 on the same model also attenuated oxaliplatin-induced cold and mechanical pain by raising the reduced action potential threshold of class-A fibers of dorsal root ganglion (DRG) neurons (30).

Considering the paclitaxel-induced peripheral neuropathic pain rat model (31), EA at GB30 reduced mechanical pain and nociceptive hypersensitivity for 3 weeks. Mechanistic studies revealed that EA may reduce CIPN symptoms by activating 5-HT1A receptors and thereby inhibiting calcium/calmodulin-dependent protein kinase II phosphorylation in the spinal cord. In the same model (32), it was found that EA at the ST36 and BL60 acupoints reduced paclitaxel-induced pain hypersensitivity. EA effectively inhibited overexpression of the transient receptor potential vanilloid 1 (TRPV1), toll-like receptor 4 (TLR4), and myeloid differentiation primary response 88 in the DRG, thus reducing TRPV1 channel activity in DRGs and inactivating spinal astrocytes and microglia. This suggests that EA may alleviate paclitaxel-induced peripheral neuropathic pain by inhibiting TLR4 signaling and upregulating TRPV1. Recent studies have indicated that antioxidant signaling in the DRG may be involved in paclitaxel-induced pathological neuropathic pain. Zhao et al. (33) found that mechanical and thermal pain thresholds were reduced by EA at PC6 and PC5 in paclitaxel-treated rats and that EA restored Nrf2-antioxidant response element/superoxide dismutase levels in the DRG and inhibited oxidative stress products 8-isoprostaglandin F2alpha and 8-hydroxy-2’-deoxyguanosine, suggesting that the inhibitory effect of EA on paclitaxel-induced nociceptive hypersensitivity may be related to the inhibition of oxidative stress in the DRG. In addition, BVA at ST36 may relieve pain by inhibiting the expression of α2 adrenergic receptors in the spinal cord to attenuate the overexcitation of spinal neurons (34). Therefore, both oxaliplatin and paclitaxel can cause inflammation and oxidative stress in the DRG and spinal cord and can induce pain in CIPN. It appears that acupoint stimulation improves peripheral neuralgia not only by acting on the 5-HT system at the site of chemotherapeutic agent accumulation but also by inhibiting the inflammatory response and oxidative stress in the area of injury.

### Chemotherapy-Induced Gastrointestinal Toxicity

Chemotherapy-induced GI includes nausea, vomiting, loss of appetite, abdominal distension, abdominal pain, diarrhea, gastrointestinal bleeding, and mucositis. These adverse reactions not only affect the quality of patient survival but also lead to poor treatment compliance and even refusal of treatment. Severe vomiting may induce dehydration, electrolyte disturbances, muscle weakness, and weight loss, even acute upper gastrointestinal bleeding, which is life-threatening. Medications such as 5-HT3 receptor and the first neurokinin 1 receptor antagonists are most commonly used in clinical practice, but while they reduce nausea and vomiting, they are ineffective in treating symptoms such as nausea, anorexia, and allotriogeusia (35, 36).

In clinical practice, 70%–80% of cancer patients receive treatment for CINV (37). Acute-phase CINV usually peaks 5 to 6 h after chemotherapy and persists for 24 h. Delayed-phase CINV usually reaches its maximum intensity 48–72 h after chemotherapeutic treatment and can last for 5 days and beyond (38). Acupoint stimulation has both preventive and therapeutic effects as an alternative therapy against delayed-phase CINV. As shown in **Table 2**, an RCT including 72 cancer patients found that TEAS at PC5 and PC6 relieved nausea and vomiting and reduced the serum levels of 5-HT and dopamine in comparison with sham TEAS (36). Li et al. (39) used EA and acupuncture to stimulate acupoints during chemotherapy in patients with advanced cancer, finding that this not only reduced the severity of chemotherapy-induced nausea and vomiting during remission but also consistently improved the nutritional status of the patients after completion of chemotherapy. LA was also effective in reducing adolescent tumor patients’ nausea scores on days 1–5 after chemotherapy, with a significant reduction in the number of vomiting episodes on days 2 and 3, suggesting that low-frequency LA is effective in relieving delayed-phase CINV-related symptoms caused by chemotherapy in cancer patients (40). In gynecological cancer, 70 patients treated with carboplatin in combination with paclitaxel found a higher rate of complete remission of delayed-phase CINV, lower nausea index, and lower incidence of adverse events such as constipation and anxiety in the group of patients who received acupuncture at PC6 before chemotherapy, suggesting that acupuncture may be effective as an alternative therapy to prevent delayed-phase CINV (41). An RCT of 60 patients treated with paclitaxel and carboplatin confirmed that the incidence of nausea, vomiting, and constipation was lower in the acupuncture group with no adverse events 2–5 days after chemotherapy, suggesting that acupuncture was better than tropisetron for the prevention and treatment of these symptoms during the delayed chemotherapy period and resulted in a better quality of life (42). In addition, daily TEAS at ST36 and PC6 after cisplatin chemotherapy in patients with liver cancer resulted in improved GI in the delayed phase, including nausea, bloating, impaired digestion, and constipation, and suggested that the mechanism may be related to vagal activity (43). Xie et al. (37) confirmed that in patients with liver cancer receiving concurrent cisplatin chemotherapy, acupoint stimulation at PC6, ST36, and LI4 improved post-chemotherapy anorexia symptoms, although significant differences were not observed in the frequency and severity of post-chemotherapy nausea and vomiting.

**Table 2:** The actions and mechanisms of acupoint stimulation in chemotherapy-induced gastrointestinal toxicity.

Clinical trials												
Ref.		Randomized type of clinical trial	Jadad score /NOS Stars	Clinical condition	Intervention		Comparison			Acupoints	Acupoint stimulation parameters	Outcomes
Zhang (36)		Yes, RCT	Jadad, 1	Cancer patients	TEAS (n = 38)		Sham TEAS (n = 34)			Bilateral: PC6, PC5	0.3 ms, 20 Hz, 10 mA, on-time of 0.1 s and off-time of 0.4 ms, 1 h, 2 times/day, 3 days	Vomiting times↓, nausea score↓, plasma levels of 5-HT and dopamine↓
Li (39)		No, single-blind sham RCT	Jadad, 4	Lung, breast, gynecologic cancer patients	Acupuncture and EA (n = 68)		Sham acupuncture (n = 66)			CV12, CV6; bilateral: LR13, ST25, PC6, ST36	Acupuncture at CV12, CV6, LR13, ST25, PC6: 10–35 mm, *deqi*, 30 min, 5 days Electroacupuncture at ST36: 2/100 Hz, 30 min, 5 days	CTCAE score↓, SNAQ score↑
Varejão (40)	Yes, RCT		Jadad, 1	Solid tumor patients	LA (n = 8)		Sham acupuncture (n = 10)			CV10, CV12; bilateral: PC6, LI4, SP6, ST36, BL20	660 nm, 30 mw, 3 joules, 1 min/point, 6 min, 5 days	Scale of the national cancer institute intensity of nausea↓
Kulthida (41)	Yes, RCT		Jadad, 4	Gynecologic cancer patients	Acupuncture (n = 35)		Ondansetron (n = 35)			Bilateral: PC6	*deqi*, 30 min, 3 weeks	Complete response rate of delayed emetic control↑, constipation and insomnia↓, cute emetic control↑, FACT-GQOL↑
Liu (42)	Yes, RCT		Jadad, 3	Gynecologic cancer patients	Acupuncture and moxibustion (n = 30)		Tropisetron hydrochloride and dexamethasone (n = 30)			CV8; bilateral: point 1	Acupuncture: 23 mm, 30 min Ginger moxibustion: ginger diameter, 5 cm, thickness, 0.5 cm, mugwort 3 cm long; 1–2 times/day, 5 min, 5 days	Vomiting times↓, nausea score↓, constipation index↓, cost↓
Zhu (43)		Yes, RCT	Jadad, 5	Liver cancer patients	TEAS (n = 37)		Sham TEAS (n = 37)			Bilateral: ST36, PC6	1 h, 2 times/week, 3 weeks; ST36: 2 s- ON, 3 s- OFF, 25 Hz, 0.5 ms, 2–10 mA; PC6: 0.25 s- ON, 0.25 s- OFF, 100 Hz, 0.5 ms, 2–10 mA	Nausea score↓, bloating scores↓, anorexia score↓
Xie (37)		Yes, RCT	Jadad, 4	Liver cancer patients	Active acupuncture (n = 72)		Placebo acupuncture (n = 70)			Bilateral: LI4, PC6, ST36	4Hz, 10mA, 30 min, 2 times/day, 6 days	VAS of anorexia score↓
Zhou (44)		Yes, RCT	Jadad, 1	Gastric cancer patients	Acupuncture (n = 28)		Control (n = 28)			Bilateral: ST36, ST37, ST25, SP6, PC6	30 min, 2 weeks	Duration of nausea↓, abdominal pain↓
Genç (45)		Yes, RCT	Jadad, 1	Breast cancer patients	Acupressure and antiemetic drugs (n = 32)		Antiemetic drugs (n = 32)			Bilateral: PC6	5 days	Index of nausea, vomiting and retching↓, beck anxiety inventory↓, mean scores for the distress↓
Basic researches												
Ref.		Model	Chemotherapeutic agents	Intervention		Acupoints		Acupoint stimulation parameters	Effect indicators		Mechanism indicators	
Baek (46)		CIA rats	Cisplatin, 6 mg/kg, a single i.p.	EA		CV12		16 mA, 10 Hz; 32 mA, 100 Hz;10 min	Body weight↑, food intake↑		(Plasma) 5-HT↑, 5-HIAA↑, DA↑, NE↑, (hypothalamus) NPY↑	
Kang (47)		CIA rats	Cisplatin, 6 mg/kg, a single i.p.	EA		CV12		10 Hz, low intensity (without muscle contractions)	Body weight↑, food intake↑		(Stomach) ghrelin mRNA↑, (hypothalamus) BDNF↑	

↑, upregulated by intervention; ↓, downregulated by intervention.

RCT, randomized controlled trial; TEAS, transcutaneous electrical acupoint stimulation; PC6, Neiguan; PC5, Jianshi; 5-HT, 5-hydroxytryptamine; EA, electroacupuncture; CV12, Zhongwan; CV6, Qihai; LR13, Zhangmen; ST25, Tianshu; ST36, Zusanli; CTCAE, common terminology criteria for adverse events; SNAQ, simplified nutritional appetite questionnaire; LA, laser acupuncture; CV10, Xiawan; LI4, Hegu; SP6, Sanyinjiao; BL20, Pishu; FACT-GQOL, functional assessment of cancer therapy-general questionnaire version; CV8, Shenque; VAS, visual analogue scale; ST37, Shangjuxu; CIA, chemotherapy-induced anorexia; 5-HIAA, 5-hydroxyindoleacetic acid; DA, dopamine; NE, norepinephrine; NPY, neuropeptide Y; BDNF, brain-derived neurotrophic factor.

Several studies have confirmed that the combination of acupoint stimulation with medication is more effective than medication alone in preventing delayed-phase CINV. For example, the results of an RCT of 56 patients treated with oxaliplatin and paclitaxel chemotherapy for advanced gastric cancer showed that acupuncture combined with esomeprazole was superior to esomeprazole alone for the treatment of gastrointestinal symptoms (nausea and vomiting, abdominal pain, and diarrhea) (44). Similarly, Genç (45) found that acupoint stimulation combined with antiemetic drugs (dexamethasone and 5-HT3 receptor antagonist and H2 receptor blocker) was more effective than antiemetic drugs alone and that acupoint stimulation also reduced anxiety and pain associated with chemotherapy, ultimately improving the quality of life of patients. Thus, for CINV induced by platinum-based chemotherapy regimens in gynecological tumors, breast cancer, liver cancer, and gastric cancer, acupoint stimulation represented by acupuncture, moxibustion, and LA is more effective in preventing and treating delayed-phase CINV, although the effects in the acute phase of CINV require further confirmation. Acupoint stimulation also has the advantages of low treatment cost, while relieving anxiety and depression and improving the quality of life, indicating that it is worthy of further clinical application.

Basic research studies on chemotherapy-induced GI have tended to focus only on CIA (46, 47). As shown in **Table 2**, Kang et al. (47) found that EA at CV12 was more effective in increasing food intake and body weight in cisplatin-treated rats, and it was suggested that EA treatment may reduce CIA by increasing the secretion of anti-anorexigenic peptides, including ghrelin and cholecystokinin. EA at CV12 and PC6 was found to alleviate cisplatin-induced nausea and vomiting by reducing the secretion of 5-HT in the duodenum and suppressing the activation of the nucleus tractus solitarius in the brain stem (48). In addition, it was (46) found that EA at CV12 not only decreased 5-TH, 5-hydroxyindoleacetic acid, dopamine, and norepinephrine but also increased appetite in rats by regulating gastrointestinal hormones and neuropeptides (ghrelin and neuropeptide Y). However, its mechanism remains to be experimentally verified.

### Chemotherapy-Induced Myelosuppression and Immunosuppression

Most cytotoxic chemotherapeutic agents will inhibit the proliferation of bone marrow (BM) hematopoietic stem/progenitor cells (HSPCs), resulting in BM hematopoietic suppression and causing chemotherapy-induced leukopenia and neutropenia. These may be the most serious side effects of chemotherapy, which may interrupt the course of antitumor treatment and induce or aggravate infections with potentially life-threatening consequences (49). The number of immune cells in the peripheral blood may also be decreased, which may lead to widespread suppression of immune cell function, resulting in overall suppression of the body’s immunity and immune escape by the tumor.

Myelosuppression in tumor patients often manifests as leukopenia or neutropenia. As shown in **Table 3**, acupoint stimulations, including TEAS, moxibustion, and EA, have been found to improve chemotherapy-induced myelosuppression and immunosuppression. An RCT of 100 patients with Lewis lung cancer (LLC) treated with gemcitabine combined with cisplatin for 28 days reported that 100 Hz TEAS at the *Dazhui* (GV14), bilateral *Geshu* (BL17), SP6, and LI4 acupoints after chemotherapy resulted in increased leukocyte counts in the peripheral blood on days 8 and 14 of chemotherapy, and increased platelet counts in the TEAS group, suggesting that high-frequency TEAS may prevent the development of chemotherapy-induced myelosuppression and may increase the number of leukocytes in the peripheral blood (50). A randomized pilot study of 18 patients undergoing chemotherapy for colorectal cancer found that six acupuncture interventions from the second to the fourth week after the start of chemotherapy were effective in boosting the peripheral blood leukocyte and neutrophil counts after 4 weeks of chemotherapy and NK cell counts in the third and fourth weeks after chemotherapy, suggesting that acupuncture has a positive immunomodulatory effect on colon cancer patients receiving chemotherapy (51). It can be concluded that acupoint stimulation can elevate peripheral leukocyte and immune cell counts, prevent the development of myelosuppression, and improve immunosuppression in cancer patients after chemotherapy.

**Table 3:** The actions and mechanisms of acupoint stimulation in chemotherapy-induced myelosuppression and immunosuppression.

Clinical trials								
Ref.	Randomized type of clinical trial	Jadad score /NOS Stars	Clinical condition	Intervention	Comparison	Acupoints	Acupoint stimulation parameters	Outcomes
Hou (50)	Yes, RCT	Jadad, 4	NSCLC patients	TEAS (n = 50)	Control (n = 50)	GV14; bilateral: BL17, ST36, SP6, LI4	6–15 V, 30/100 Hz, 30 min/day, 9 days	WBC count↑, platelet count↑, degree of comfort↑
Irene (51)	Yes, RCT	Jadad, 3	CRC patients	Acupuncture and moxibustion (n = 9)	Control (n = 9)	CV6, bilateral: LR3, ST36, SP3, GB39, LI4, PC5, LU7, SI6, ST32	Acupuncture: 10 mm, *deqi*; moxibustion: SI6, ST32, CV6, 2 min/point, 45 min, 2 times/week, 6 sessions	WBC count↑, ANC↑, NK cells↑, HADS↓

↑, upregulated by intervention; ↓, downregulated by intervention.

RCT, randomized controlled trial; NSCLC, non-small cell lung cancer; TEAS, transcutaneous electroacupuncture stimulation; GV14, Dazhui; BL17, Geshu; ST36, Zusanli; SP6, Sanyinjiao; LI4, Hegu; WBC, white blood cell; CRC, colorectal cancer; CV6, Qihai; LR3, Taichong; SP3, Taibai; GB39, Xuanzhong; PC5, Jianshi; LU7, Lieque; SI6, Yanglao; ST32, Futu; ANC, absolute neutrophil count; NK cell, natural killer cell; HADS, hospital anxiety and depression scale.

Using stimulation at the GV14, BL17, ST36, and *Shenzhu* (BL23) acupoints in mice (52, 53), acupuncture and moxibustion were found to increase the numbers of peripheral blood leukocytes, the expression of CyclinD1 in BM cells, and the percentages of S-phase and G2/M cells in the BM cell cycle in mice with chemotherapy-induced myelosuppression, suggesting that acupoint stimulation promotes hematopoiesis by accelerating the transition of cells from the G1 to S phase and increasing DNA synthesis. The sympathetic projection is the most critical factor orchestrating BM cell proliferation, differentiation, and egress (54, 55). We previously reported that neuropeptide pituitary adenylate cyclase-activating polypeptide (PACAP) is secreted by sympathetic nerve endings projected into the BM and can modulate HSPC proliferation *via* PACAP-specific receptor (PAC1) signaling (56). Our recent study has shown that EA on ST36 and SP6 prevents neurotoxicity, preserves BM hematopoiesis, and myeloid ontogenesis during cisplatin chemotherapy. EA induced both neuro and immune protection by inducing the neurogenic production of PACAP, which preserves BM hematopoiesis *via* the PAC1 receptor (57).

The BM is the largest organ controlling immunity, and chemotherapeutic agents causing myelosuppression may also lead to simultaneous immunosuppression (58, 59). In response to immunosuppression in breast cancer mice caused by paclitaxel chemotherapy, moxibustion at ST36 combined with paclitaxel significantly increased the spleen and thymus indices and peripheral blood leukocyte counts in tumor-bearing mice, thereby improving their immune function. Moxibustion also mitigated the weight loss and improved the survival rate of the cancer-bearing mice. By measuring the serum levels of cytokines, it was further confirmed that moxibustion combined with paclitaxel could upregulate interferon-γ (IFN-γ) and interleukin-2 (IL-2) and downregulate IL-10 and transforming growth factor-β to enhance immune function (60). Our previous study also showed that cisplatin inhibited all leukocyte subpopulations and was more detrimental to T lymphocytes than to B lymphocytes in xenograft mice. This indicates the potential of cisplatin to induce immunosuppression and limit antitumor immune responses (61). Based on the fact that acupoint stimulation can improve myelosuppression and immunosuppression caused by cisplatin, and BM hematopoiesis is an important source of immune cell subgroups in the tumor immune microenvironment (TIME), we speculate that acupoint stimulation may contribute to the inhibition of tumor growth and the improvement of the TIME *via* recovery of BM hematopoiesis.

## The Efficacy and Mechanism of Acupoint Stimulation on Tumor Growth

Currently, there are few clinical studies on the use of acupoint stimulation to increase the antitumor effect of chemotherapy. As shown in **Table 4**, only one RCT of 53 patients with advanced squamous cervical cancer reported four cycles of chemotherapy treatment with patients receiving EA at the bilateral ST36 acupoints, it was found that EA treatment reduced the cervical tumor volume and increased NK cell counts in the peripheral blood, suggesting that EA may exert its antitumor effect by increasing the numbers of the body’s NK cells. In addition, EA can increase the efficacy of chemotherapy and inhibit tumor growth, while at the same time alleviating chemotherapy-induced pain, appetite, nausea, and vomiting (14). Nevertheless, more clinical trials are necessary for verification.

**Table 4:** The actions and mechanisms of acupoint stimulation in anticancer effect.

Clinical trials										
Ref.	Randomized type of clinical trial	Jadad score /NOS Stars	Clinical condition	Intervention		Comparison	Acupoints	Acupoint stimulation parameters	Outcomes	
Saraswati (14)	Yes, RCT	Jadad, 1	SCC patients	EA (n = 28)		Control (n = 25)	Bilateral: ST36	10–15 mm, 0.2 mA, 2 Hz, 30 min	Percent of NK cells↑, tumor size↓	
**Basic researches**										
**Ref.**	**Model**	**Chemotherapeutic agents**	**Intervention**		**Acupoints**	**Acupoint stimulation parameters**	**Effect indicators**			**Mechanism indicators**
Behranvand (60)	Breast cancer nude mice	Paclitaxel, 1mg/ml	Moxibustion		ST36	3 pillars/acupoint, 1 time/day	Tumor volume↓, body weight↑, survival↑, histological analysis↑, thymus index↑, spleen index↑, WBCs↑, IL-2↑, IFN-γ↑, IL-10↑, TGF-β↑			CD34↓, HIF-1α↓,VEGFA↓,PD-1↓,PD-L1↓
Yang (62)	Breast cancer nude mice	Paclitaxel	EA		4 needles were inserted toward the tumor at up, down, left and right directions in tumor-bearing mice	Low frequency: 3–4 Hz, 5 s; high frequency: 15–20 Hz, 10 s, longitudinal wave setting	Tumor volume↓, fluorescence images↑, intratumoral paclitaxel concentration↑, tumor cell apoptosis↑			CD34↓, COL1V↑, α-SMA↑, NM23↑
Xu (63)	Osteosarcoma mice	—	Acupuncture		BL23, GV20, ST36	1 time/day, 2 weeks	Tumor volume↓, body weight↑, T cell↓, NK cell↓, toxic T cells↓			IL-6↓, IFN-γ↓, TGF-α↓
Smeester (64)	Osteosarcoma mice	—	EA		ST36	4 Hz pulse rate, 100 µs pulse width, 30 min, 2times/week	Tumor growth and lung metastasis↓, tumor lymphatics, vasculature, and innervation↓			—
Lee (65)	CRC rats	Azoxymethane	PN		HT7, SI5	4 weeks, everyday	Aberrant crypt foci↓, tumor mass↓, number size↓			—
Wang (61)	LLC mice	Cisplatin, 3 mg/kg,2 times/week, 2 weeks	Moxibustion		ST36	15 min, 5 times/week, 2 weeks	Body weight↑, tumor volume↓, tumor area↓, T cells↑ (CD8+T, CD4+T, Th1), myeloid cells (M1) ↑, CD69↑, INF-γ↑, CD86↑			CD31↑, α-SMA↑,VEGF↓

↑, upregulated by intervention; ↓, downregulated by intervention.

RCT, randomized controlled trial; SCC, cervical squamous cell carcinoma; EA, electroacupuncture; ST36, Zusanli; NK cell, natural killer cell; WBC, white blood cells; IL-2, interleukin-2; IFN-γ, interferon-γ; IL-10, interleukin-10; TGF-β, transforming growth factor β; HIF-1α, hypoxia inducible factor-1α; VEGFA, vascular endothelial growth factor A; PD-1, programmed death-1; PD-L1, PD-1 ligand; COLIV, collagen IV; α-SMA, α-smooth-muscle actin; NM23, non-metastasis-23; BL23, Shenshu; GV20, Baihui; IL-6, interleukin-6; TGF-α, transforming growth factor α; CRC, colorectal cancer; PN, nanoporous acupuncture needle; HT7, Shenmen; SI5, Yanggu; LLC, Lewis lung cancer; VEGF, vascular endothelial growth factor.

Basic research on the tumor-inhibiting effects and mechanisms of acupoint stimulation has also been conducted. In this review, we retrieved seven basic studies, which showed that acupoint stimulation with chemotherapy could delay tumor growth and metastasis to some extent in mice with breast cancer (60, 62), osteosarcoma (63, 64), colon cancer (65), and non-small cell lung cancer (61) models and could prolong survival in mice (shown in **Table 4**).

The systemic distribution of chemotherapeutic agents is an important cause of the multiple side effects of post-tumor chemotherapy (66); moreover, long-term repeated use of chemotherapeutic agents will inevitably lead to a decrease in the sensitivity of the drugs and may increase the risk of tumor resistance and the possibility of tumor metastasis. Therefore, it is important to improve the specific targeting of chemotherapeutic agents to tumor tissues and cells to eliminate the tumors. Yang et al. (62) administered paclitaxel at a dose of 10 mg/kg intravenously to breast cancer tumor-bearing mice and observed whether EA could alter the tumor microenvironment (TME) to guide the delivery of the chemotherapeutic agents to the breast cancer region. Four needles were inserted toward the tumor in up, down, left, and right directions to increase the peak paclitaxel concentration. TdT-mediated dUTP Nick-End Labeling analysis showed that the combination of EA with paclitaxel treatment was more likely to increase apoptosis in the tumor cells. In addition, the levels of α-smooth muscle actin and collagen IV (COLIV) were higher and hypoxia-inducible factor-1α levels were downregulated in the combined EA-chemotherapy treatment group. It is thus concluded that local EA intervention can increase the localized concentration of the chemotherapeutic agent in tumors, which may be the result of alterations in the TME.

Tumor cells can trigger multiple immunosuppressive cascade responses, including the induction of immature immune cells to differentiate into immunosuppressive phenotype cells (67). Meanwhile, vascular abnormalities and dysfunction within solid tumors can lead to hypoxia, glycolysis, low pH, high vascular permeability, and interstitial pressure (68), which impair the infiltration of antitumor immune effector cells and chemotherapeutic agents into the tumor tissue (69). On the contrary, changes in the cellular and molecular dynamics of the TME can lead to the accumulation of large numbers of immunosuppressive cells and inflammation-related factors, promoting proliferation, immune escape of tumor cells, and metastasis (70). Therefore, attenuating immunosuppression and vascular abnormalities may be a strategy to improve the outcomes of chemotherapy in cancer patients.

Our previous study (61) used moxibustion at the ST36 acupoint combined with cisplatin chemotherapy in LLC mice and found that the tumor-suppressive effect of moxibustion combined with cisplatin chemotherapy was superior to that of chemotherapy alone or moxibustion alone. It was further observed that moxibustion upregulated the infiltration of CD4^+^ T cells and Th1 cells in tumors and that the combination therapy increased the expression of CD8^+^ cytotoxic T cells, CD4^+^ T cells, Th1, Th9 cells, and M1 macrophages and genes expressing CD69, IFN-γ, and CD86. In addition, moxibustion combined with cisplatin improved the tumor vascular architecture by increasing cell coverage and promoted tumor vascular normalization by inhibiting the expression of angiogenic factor vascular endothelial growth factor (VEGF). These findings indicate that moxibustion combined with cisplatin treatment could inhibit LLC tumor growth and that the effects involve the improvement of the tumor immune-vascular microenvironment. Xue et al. (60) also found reduced weight loss and improved survival rates in mice with breast cancer by administering a single tail vein injection of the chemotherapeutic agent paclitaxel at a dose of 1 mg/ml and treating the mice with moxibustion at the ST36 acupoints bilaterally for 2 weeks. Moxibustion combined with paclitaxel also enhanced immune function by upregulating IFN-γ and IL-2, downregulating IL-10 and transforming growth factor-β1, and increasing the leukocyte count and the thymic and splenic indices. Notably, moxibustion combined with paclitaxel inhibited tumor angiogenesis by downregulating CD34, hypoxia-inducible factor-1α, and VEGFA and counteracted the immunosuppressive microenvironment by inhibiting the interactions between programmed death-1 (PD-1) and its ligand PD-L1. These findings also suggest that ST36 stimulation by moxibustion can promote the antitumor effect of chemotherapy associated with alleviation of the immunosuppressive microenvironment and the promotion of vascular normalization. However, the detailed molecular mechanisms require further investigation.

Some studies have also found that acupoint stimulation can inhibit tumor growth directly. Jiang et al. (71) found that 4T1-transplanted triple-negative breast cancer-bearing mice treated with EA for 3 weeks showed reduced tumor sizes, with the effects on tumor growth increasing with the duration of the treatment. Meanwhile, EA has been shown to reduce tumor expression of VEGFA and its receptor VEGFR and to downregulate the expression of neuropilin 1. The underlying mechanism may be related to the sharp decline of angiogenic factors (VEGFA, VEGFR, and neuropilin 1), the reduction in extracellular matrix components (metalloproteinase-2 and tissue inhibitor of matrix metalloproteinase), triple-negative breast cancer-induced inflammation [tumor necrosis factor-alpha (TNF-α)], and an increase in nerve growth factor receptors (nerve growth factor p75 and semaphorin 3A).

In summary, it is apparent that the mechanism of acupoint stimulation (EA and moxibustion) involves synergy with chemotherapy to inhibit tumor growth (breast cancer, non-small cell lung cancer) and may be related to the alleviation of the immunosuppressive microenvironment and the promotion of vascular normalization, further increasing the concentrations of chemotherapeutic drugs in tumor tissue to restore the overall functional state of the TME and the whole body.

## Discussion and Conclusion

### The Benefits of Acupoint Stimulation Combined With Chemotherapy in Cancer Patients

**Figure 1:**
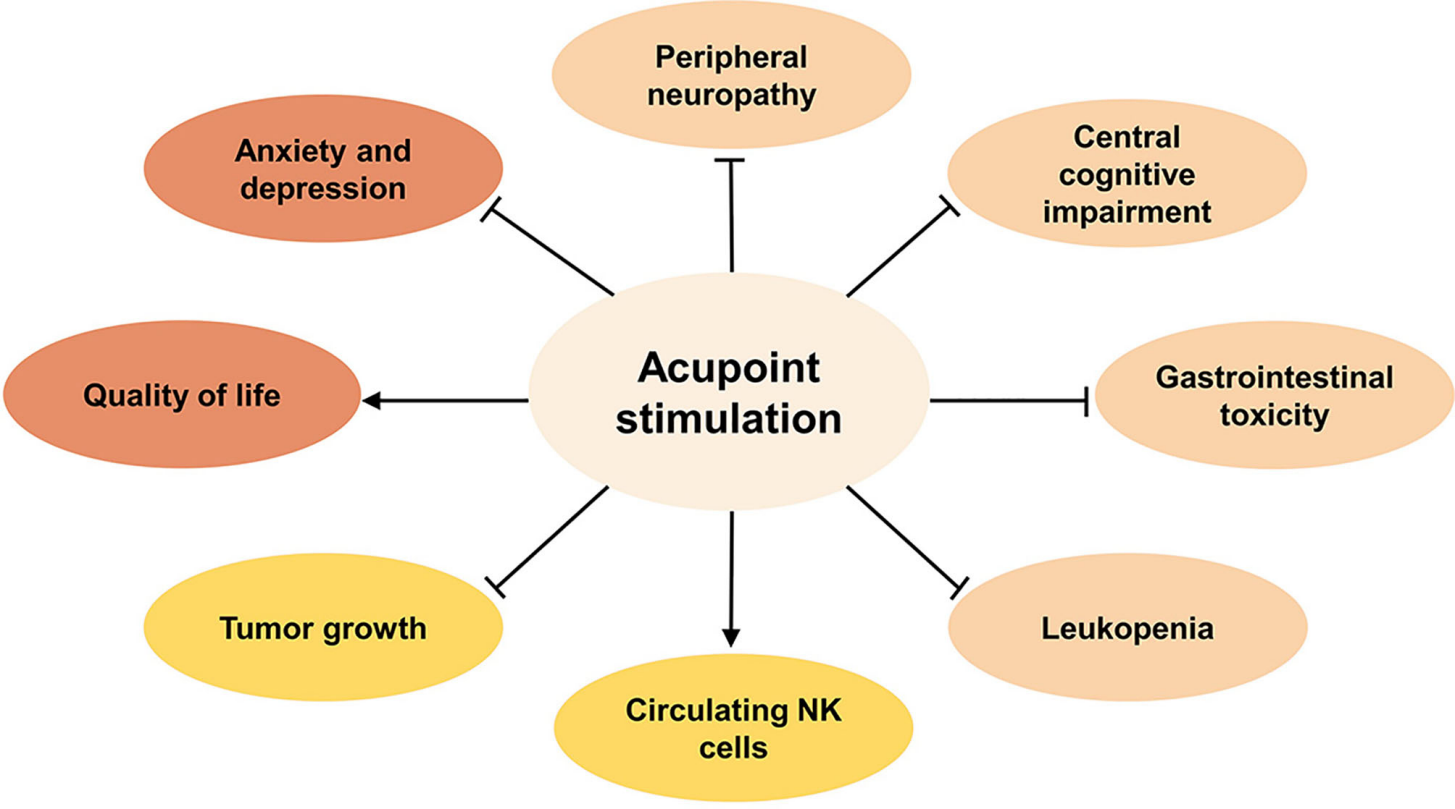
The benefits of acupoint stimulation combined with chemotherapy in cancer patients. Inhibit ⊣, Promote →.

To summarize the current clinical evidence (**Figure 1**), acupoint stimulation improved clinical symptoms induced by chemotherapy toxicity, including peripheral neuropathy such as neuropathic pain and numbness, central cognitive impairment such as reduced decreased memory and learning ability, GI such as nausea and vomiting, or leukopenia. In addition, negative emotions such as anxiety and depression that accompanied chemotherapy toxicity, as well as the patients’ health outlook, including overall quality of life and nutritional status, were found to have improved significantly after treatment.

These effects contribute to the survival expectations of oncology patients and coincide with the purpose of palliative care. Although acupoint stimulation still needs to be incorporated into the cancer palliative care system, we cannot ignore the palliative effect of acupoint stimulation on cancer. For instance, acupoint stimulation is useful not only to reduce the side effects of chemotherapy but also to relieve tumor-related symptoms (including fatigue, insomnia, and cancer pain) and relieve tumor treatment-related side effects [including constipation caused by opioid peptides (72), lymphedema after breast cancer surgery (73), and knee pain caused by aromatherapy enzyme inhibitors (74, 75)]. Taking cancer pain as an example, acupoint stimulation is beneficial for patients with moderate to severe cancer pain. The use of acupoint stimulation has also been written into a number of clinical and evidence-based medical guidelines (76), indicating that acupoint stimulation can be used as an important form of palliative therapy as part of the comprehensive treatment and management of cancer pain in patients. In addition, the ability of acupoint stimulation to regulate both the body’s immunity and the TME (77) indicates that acupoint stimulation has the potential for development into an effective treatment for targeting the TME to combat cancer.

There are several limitations to the clinical evaluation in this review. On one hand, nine of 18 included RCTs were of high quality with Jadad scores ranging from 4 to 6, while the remaining nine studies were of low quality with Jadad scores between 1 and 3 (**Tables 1** **–** **4**). The reasons for the low-quality designation included the following: (1) despite mentioning the word “random,” the studies did not describe the random sequence generation method in detail, thus increasing the risk of selection bias; (2) most studies were not double-blinded, so it is difficult to guarantee the authenticity of the results; (3) most studies did not mention or describe in detail the reasons for withdrawal and loss to follow-up, resulting in low authenticity and reliability. The included cohort study was of moderate quality (five stars in Newcastle–Ottawa scale, **Table 1**). On the other hand, since this review focused on multiple side effects and tumor-inhibitory effects of chemotherapy, systematic evaluation could not be applied. There was poor consistency between the effect indicators and the quality of the included studies, resulting in a failure of the quantitative analysis of the total effect size; thus, the results should be interpreted cautiously due to the small number and low quality of the included studies. More high-quality clinical studies are required to confirm the clinical value of acupoint stimulation. To date, several systematic reviews and meta-analyses have indicated that the use of acupuncture in the management of CIPN (78), CINV (79), and chemotherapy-induced leukopenia (80) is safe and effective. However, there are few clinical studies on the effects of acupoint stimulation on chemotherapy-induced myelosuppression and immunosuppression nor on tumor growth.

### Neuroimmune Regulation Involved in the Alleviation of Chemotherapy-Induced Side Effects by Acupoint Stimulation and Its Role in Assisting the Anticancer Action of Chemotherapy

Neuroinflammation is responsible for many of the side effects of chemotherapy. For example, the accumulation of chemotherapeutic agents in the DRG of animals leads to the production and buildup of inflammatory cells and pro-inflammatory cytokines, which, in turn, damage the DRG neurons and consequently lead to peripheral neuropathy. Macrophages play key roles in the enhancement of pain and can cause peripheral sensitization (81, 82). In addition, peripheral sensitization and the prolonged abnormal transmission of pain signals to the dorsal horn of the spinal cord result in increased excitability of neurons at the site of the pain, as well as glial cell activation triggering central sensitization (83–85). The inflammatory response in neural tissue includes immune cell accumulation and cytokine/chemokine release, e.g., paclitaxel directly activates TLR4 and its downstream pathways, leading to an accumulation of macrophages and T cells in neural tissue and the release of large amounts of pro-inflammatory cytokines (TNF-α, IL-17, IL-20, CCL2) *via* DRG neurons or associated cells by modulating the expression and activity of ion channels in neurons and spinal cord receptors (86).

The chemotherapeutic agent doxorubicin is known for generating reactive oxygen species, leading to oxidative stress in the tumor and even throughout the body (87–89). Increased levels of peripheral oxidative stress elevate the levels of circulating TNF-α, cross the blood–brain barrier, activate locally produced pro-inflammatory cytokines such as IL-6 and IL-1β, and raise the levels of oxidative stress in the brain (90–93). The resulting combination of neuroinflammation and oxidative stress can cause apoptosis and neurobehavioral changes. Dose-dense paclitaxel treatment leads to a 7-fold higher concentration of the drug in hippocampal tissue than in the neocortex, suggesting that neuroinflammation may mediate chemotherapy-induced cognitive impairment (94). Most chemotherapeutic agents damage the overall gastrointestinal tract, leading to mucosal inflammation (95). In addition, the maintenance of inflammatory signaling homeostasis plays a complex role in hematopoietic stem cell maintenance (96), activation, proliferation, and differentiation, while exposure to acute or chronic inflammation can have deleterious effects on hematopoietic stem cell hematopoiesis and self-renewal. Although chemotherapeutic agents can directly inhibit BM hematopoietic stem cell function, sympathetic neurotoxic injury *via* the BM also contributes to BM suppression and leukopenia (57). Thus, chemotherapy-induced cognitive impairment, neuropathic pain, adverse GI, and BM suppression may share a common pathological mechanism, specifically, neuroinflammation.

A significant amount of evidence suggests that acupoint stimulation is effective in inflammatory diseases, such as asthma, allergic rhinitis, inflammatory bowel disease, and rheumatoid arthritis. The anti-inflammatory mechanism is mainly focused on the inhibition of pro-inflammatory factors and the promotion of anti-inflammatory factors released by acupuncture through the induction of macrophage polarization. Through systematic research, our team has found that needles can activate the autonomic nervous system to secrete specific neuromodulators, which further induce macrophage polarization directly or indirectly by regulating T-cell polarization-related cytokines in the local inflammatory environment (82). In recent years, acupuncture has shown remarkable results in counteracting inflammation through the vagal and sympathetic nervous systems. For example, EA can reduce sepsis-induced damage to multiple internal organs such as the lung, heart, kidney, liver, and gastrointestinal tract (97). The mechanism is that EA stimulates peripheral nerves to induce somatic-autonomic reflexes that, in turn, regulate the functions of the corresponding organs. Based on the law of spinal cord segmental control of spinal somatic-sympathetic reflexes, it is hoped that more rigorous experiments can be designed in the future to demonstrate whether acupuncture can holistically modulate tumor-induced multisystem damage by needling acupoints in specific nerve segments. In addition, EA increases vagal nerve activity, suppresses systemic inflammation, and attenuates pancreatic histopathological changes and leukocyte infiltration (98). In the case of sepsis, the neuroanatomical basis of the EA-driven vagus–adrenal axis is becoming clearer after the *Nature Medicine* report that EA of the sciatic nerve can lead to dopamine production in the adrenal medulla and can control systemic inflammation through activation of the vagus nerve (85, 99–101). However, the neuro-immune regulatory mechanisms mediating the acupoint stimulation alleviation of chemotherapy-induced side effects require more investigation.

Acupoint stimulation reduces immunosuppression in the TME, vascular abnormalities, and inflammatory progression. Regulation of macrophage polarization toward antitumor M1 macrophages is an effective way to overcome the TME immunosuppression and inhibit tumor growth and immune escape (102, 103). Based on previous studies, EA at ST36 and SP6 promotes the proliferation of BM HSPCs through repairing BM sympathetic nerves, releasing PACAP *via* receptor PAC1, and ameliorating chemotherapy-induced peripheral leukopenia. It is suggested that acupuncture may promote increased proliferation of BM HSPCs, increasing the production of peripheral leukocytes that may contribute to promoting the organic immune function and TIME.

Our previous study found that moxibustion can upregulate IFN-γ concentrations and Th1 and M1 populations. IFN-γ secreted by Th1 cells stimulates the activity of M1 tumor-associated macrophages, which could inhibit tumor growth (104, 105). IFN-γ secreted by Th1 cells plays a critical role in the “immune reprogramming and vascular normalization loop” (106) and may alter the expression of CXCL9, CXCL10, CXCL11, and CXCL11 by localizing in the vicinity of vascular endothelial cells. These chemokines regulate pericyte recruitment and attachment, resulting in vascular normalization. Vascular normalization, in turn, alters the immune microenvironmental landscape by recruiting T lymphocytes and reducing neutrophil levels. At the molecular level, this mutually regulated loop is coordinated in part by IFN-γ and CD40^+^T through their actions on the immune response. Activation of this loop not only enhances antitumor effects but ultimately promotes immune-mediated tumor eradication, and interruption or failure to establish this positive reinforcement process may lead to rapid drug resistance and rapid tumor progression (107). We speculate that moxibustion combined with chemotherapy can control tumor progression by modulating vascular and immune crosstalk but needs to be confirmed by further studies.

**Figure 2:**
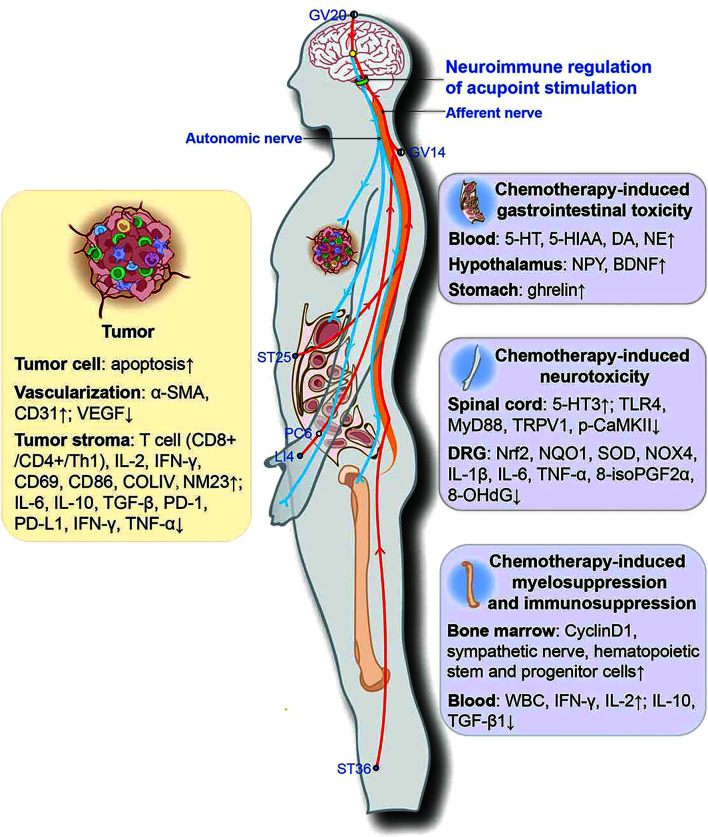
Potential neuroimmune regulatory mechanisms involved in acupoint stimulation combined with chemotherapy for combating cancer. ●, the outer side of the body; ◐, the dorsoventral midline of the body; ○, the medial side of the body. α-SMA, α-smooth-muscle actin; VEGF, vascular endothelial growth factor; COLIV, collagen IV; NM23, non-metastasis-23; PD-1, programmed death-1; PD-L1, programmed death-1 ligand; 5-HT, 5-hydroxytryptamine; 5-HIAA, 5-hydroxyindoleacetic acid; MyD88, myeloid differentiation primary response protein 88; p-CaMKII, phosphorylated calcium/calmodulin-dependent protein kinase II; Nrf2, nuclear factor (erythroid-derived 2)-like 2; NQO1, NADPH quinone oxidoreductase-1; SOD, superoxide dismutase; NOX4, NADPH oxidase 4; 8-isoPGF2α, 8-isoprostane; 8-OHdG, 8-hydroxy-2-deoxyguanosine.

In conclusion, current research on acupuncture mechanisms shows that acupoint stimulation improves chemotherapy-induced peripheral neuropathy and GI by modulating the 5-HT system in the dorsal root ganglia, the dorsal horn of the spinal cord, and the duodenum by reducing oxidative stress and neuroinflammation. Acupoint stimulation also alleviated GI by activating the vagus in the nucleus of the tractus solitarius and promoting the secretion of gastrointestinal hormone neuropeptides. Acupoint stimulation also restored bone marrow hematopoiesis and the body’s immune function to combat cancer. It is suggested that acupoint stimulation may repair the sympathetic nerves in the bone marrow damaged by chemotherapeutic agents and promote the proliferation of bone marrow hematopoietic stem and progenitor cells. Meanwhile, acupoint stimulation combined with chemotherapy could inhibit tumor growth by promoting tumor cell apoptosis and increasing the accumulation of chemotherapeutic agents in tumor tissues, as well as by modulating the TIME and vascular normalization. Accumulating evidence also indicates that neuroimmune regulation may be involved in these actions (**Figure 2**). This evidence suggests that acupoint stimulation can both alleviate the side effects of chemotherapeutic agents and assist the drugs in reducing tumor growth, thus expanding the clinical application of acupoint stimulation in cancer treatment. Accumulating evidence also indicates that neuroimmune regulation may be involved in these actions; however, systematic mechanistic studies are required for verification.

## Author Contributions

ZX, YC, and YG: conceptualization and supervision. ZX, YC, SLi, and SZ: methodology, data collection and article writing. YY and JH: data collection and analysis. JW and SLu: preparation of the figures and the graphical abstract. YC: project administration. CC and BW: funding acquisition. All authors contributed to the article and approved the submitted version.

## Funding

This work was financially supported by the National Natural Science Foundation of China (grant numbers 81901728, 82030125, 82074534).

## Conflict of Interest

The authors declare that the research was conducted in the absence of any commercial or financial relationships that could be construed as a potential conflict of interest.

## Publisher’s Note

All claims expressed in this article are solely those of the authors and do not necessarily represent those of their affiliated organizations, or those of the publisher, the editors and the reviewers. Any product that may be evaluated in this article, or claim that may be made by its manufacturer, is not guaranteed or endorsed by the publisher.

## Abbreviations

NSCLC, non-small cell lung cancer; CIPN, chemotherapy-induced peripheral neuropathy; CICI, chemotherapy-induced cognitive impairment; CINV, chemotherapy-induced nausea and vomiting; CIA, chemotherapy-induced anorexia; EA, electroacupuncture; TEAS, transcutaneous electrical acupoint stimulation; BVA, bee venom acupuncture therapy; LA, laser acupuncture; RCT, randomized controlled trial; NK cell, natural killer cell; GI, gastrointestinal toxicity; BDNF, brain-derived neurotrophic factor; 5-HT, 5-hydroxytryptamine; DRG, dorsal root ganglion; TRPV1, transient receptor potential vanilloid 1; TLR4, toll-like receptor 4; BM, bone marrow; HSPCs, hematopoietic stem/progenitor cells; PACAP, pituitary adenylate cyclase-activating polypeptide; PAC1, PACAP-specific receptor; IFN-γ, interferon-γ; IL, interleukin; TIME, tumor immune microenvironment; TME, tumor microenvironment; LLC, Lewis lung cancer; VEGF, vascular endothelial growth factor; TNF, tumor necrosis factor; HSC, hematopoietic stem cell.
